# Blink parameters are confounded by vertical eye orientation in video-based eye tracking: Comparing pupil- and eyelid-based methods

**DOI:** 10.3758/s13428-026-02984-4

**Published:** 2026-03-20

**Authors:** Wolf Culemann, Ignace T. C. Hooge, Diederick C. Niehorster, Angela Heine, Marcus Nyström

**Affiliations:** 1 Institute of Psychology, University of Duisburg-Essen, Universitätsstr. 2, Essen, 45141 Germany; 2 Experimental Psychology, Helmholtz Institute, Utrecht University, Utrecht, The Netherlands; 3 Humanities Lab, Lund University, Lund, Sweden; 4 Department of Psychology, Lund University, Lund, Sweden

**Keywords:** Eye blinks, Blink characteristics, Blink duration, Vertical eye orientation, Eye openness, Eye tracking

## Abstract

Blink characteristics such as duration, amplitude, and eyelid velocity are widely used indicators of cognitive and physiological states. While early magnetic search coil studies suggested that vertical eye orientation relative to the head influences blink measurements, subsequent research has largely ignored this factor. No studies have investigated whether vertical eye orientation effects replicate in modern video-based methods or whether different video-based blink-detection approaches show similar sensitivities to changes in vertical eye orientation. In this study, we investigated how vertical eye orientation affects blink parameters estimated using both pupil-based and eyelid-based detection. We recorded pupil diameter and estimated eye openness from video data as seventeen participants performed voluntary blinks from three vertical eye orientations while keeping their heads stationary. Vertical eye orientation systematically influenced all measured blink parameters. Eye openness at blink onset and closing amplitude decreased with downward eye orientation. With more downward eye orientation, closing velocity increased, whereas opening velocity decreased. Crucially, pupil-based measurements of blink duration showed much larger vertical eye orientation effects than measurements of eye openness (32% vs. 8% increase of blink duration from upward to downward eye orientation), though eyelid-based estimates are sensitive to how blink onset and offset are derived. These results show that the vertical eye orientation is a systematic confounding factor in video-based blink measurement, with the measurement method influencing the magnitude of observed effects. The findings have important implications for studies investigating blink characteristics where vertical eye orientation varies, and we conclude with practical recommendations for study design and reporting.

## Introduction

Blinks – the rapid closure and reopening of the eyelids – are widely used behavioral markers across cognitive, clinical, and applied domains given that blink characteristics (e.g., duration, amplitude, and eyelid velocity) are sensitive to physiological and cognitive state (Stern et al., 1984). Estimated blink parameters are commonly used as indicators of fatigue (Caffier et al., 2003; Cori et al., 2019) and attentional state (Benedetto et al., 2011; D’Mello et al., 2017; Hollander & Huette, 2022; Oyarzo et al., 2022), and are increasingly used in applied monitoring systems (e.g., driver monitoring Benedetto et al., 2011; Cori et al., 2019).

Earlier studies investigating blinking behavior employed magnetic search coils and electromyography (Stern et al., 1984; Vanderwerf et al., 2003). Magnetic search coils taped to the eyelid detect orientation changes within a magnetic field, while electromyography records electrical activity of the muscles controlling the eyelids. While especially magnetic search coils offer high precision measurement of eyelid orientation, they are invasive and largely limited to laboratory settings.

However, contemporary research and the majority of applied studies rely on video-based eye-tracking for blink detection and characterization due to the non-invasive nature and broad accessibility of this method. Video-based methods to quantifying blinks generally fall into two categories: The first is pupil-based and is widely used in commercial eye trackers due to its simplicity and computational efficiency (Culemann et al., 2023; Hoogerbrugge et al., 2022; Nyström et al., 2024). Blinks are here typically defined as periods of missing pupil data, sometimes extended based on sharp changes in pupil size immediately before and after pupil data loss (Culemann et al., 2023; Hershman et al., 2018). The second approach involves direct tracking of eyelid position and deriving metrics like eye openness – the distance between the upper and lower eyelid (Nyström et al., 2024) – or the eye-aspect-ratio, which calculates the ratio between eye height and width based on eyelid landmarks (Soukupova & Cech, 2016). These methods can provide richer kinematic information but have only recently become available in some commercial eye tracking systems (Nyström et al., 2024). Recent methodological studies demonstrate that blink parameters estimated from pupil and eyelid positions are not interchangeable, as they can differ systematically in terms of, for instance, timing, duration, and amplitude of blinks (Culemann et al., 2023; Nyström et al., 2024).

A critical insight from early magnetic search coil studies is that vertical eye-in-head orientation – hereafter referred to as vertical eye orientation – systematically modulates blink characteristics. Stern et al. (1984) described that upward eye orientation can increase blink amplitude due to the elevated starting position of the upper eyelid, while downward eye orientation may attenuate blink amplitude. Vanderwerf et al. (2003) empirically demonstrated these effects in four subjects, showing that upward eye orientation resulted in larger closing amplitudes, shorter blink durations, and higher maximum closing velocities, whereas downward eye orientation produced slower, longer blinks with reduced amplitude. Vanderwerf et al. (2003) showed systematic variations across different vertical eye orientations, with blink durations being consistently shorter in upward eye orientation (121–163 ms) than in downward eye orientation (154–203 ms). Notably, no such effects were observed for horizontal eye orientation.

Despite these early insights, subsequent blink research has largely ignored vertical eye orientation as a potential confound. Many contemporary studies using video-based methods do not control for or report vertical eye orientation (Benedetto et al., 2011; Hollander & Huette, 2022; Oyarzo et al., 2022), potentially introducing an unrecognized confound when estimated blink parameters are interpreted as markers of fatigue, attention, or cognitive state. This oversight is particularly problematic given that many applied contexts – such as reading or driving studies – involve systematic variations in vertical eye orientation that thus could systematically bias blink measurements. For instance, the natural progression from looking at text at the top to the bottom of a page in reading studies, or shifts between monitoring the road ahead versus dashboard instruments in driving contexts, may produce apparent changes in blink characteristics that in fact reflect changes in vertical eye orientation rather than changes in cognitive or physiological state. Such confounds could lead to misinterpretation of research findings or unreliable performance in applied monitoring systems.

Importantly, the field lacks direct evidence under the measurement conditions it now primarily relies on – video-based eye tracking – regarding potential biases from vertical eye orientation. Accordingly, whether – and how – vertical eye orientation biases different blink-detection approaches remains an open question. In this paper, therefore, we directly test whether effects reported with magnetic search coils replicate when blinks are measured with modern video-based eye trackers. Moreover, because the two dominant video approaches – pupil-based and eyelid-based – rest on different detection principles (pupil occlusion vs. eye openness), vertical eye orientation could influence blink parameters to different degrees for these two methods. Clarifying whether and how vertical eye orientation influences video-based blink estimation, and whether such influence differs across detection methods, is essential for valid and robust blink studies.

### Aims and hypotheses

We systematically investigated whether vertical eye orientation affects blink amplitude, duration, and peak velocities in contemporary video-based measurements, testing both: (a) pupil-based, and (b) eyelid-based detection. Our primary, overarching question is:

*How does vertical eye orientation affect blink parameters estimated from data recorded with video-based eye trackers, and do these effects differ between pupil-based and eyelid-based methods?*

Based on prior work with magnetic search coils tracking the eyelids (Vanderwerf et al., 2003) we predict that video-based eyelid blink parameters will show the pattern: *upward eye orientation is associated with larger closing amplitude, shorter blink duration and higher closing velocity*, compared with *downward eye orientation, that is associated with smaller amplitude, longer blink duration and lower closing velocity*. For pupil-based blink parameters, we predict similar directional effects but potentially different magnitudes because this method depends on the relative geometry of the pupil and eyelids rather than the eyelid trajectory. Effects on opening velocity are less well documented in prior work and are therefore treated as exploratory.

## Methods

We used a within-subjects design and recorded voluntary blinks at three vertical eye orientations while keeping the head position stationary. We measured pupil size with an established eye tracker (the EyeLink 1000 Plus) and estimated eye openness from co-recorded high-speed video using custom algorithms.

### Participants

Seventeen participants (13 female, four male; age range, 20–57 years) completed the study at Freie Universität Berlin (FUB; *n* = 8) and University of Duisburg-Essen (UDE; *n* = 9). Two additional participants were excluded early in data collection due to pupil detection interference at the bottom target position, which was resolved for subsequent participants by slightly lowering the eye tracker setup. All participants completed the experiment without glasses (contact lenses permitted). Four participants reported left eye dominance and 13 participants reported right eye dominance. One participant wore contact lenses. The study was approved by the Ethics Committee of the University of Duisburg-Essen (EA-PSY16/25/250420254), and all participants provided written informed consent.

### Apparatus

#### Pupil size and gaze tracking

An EyeLink 1000 Plus (SR Research) recorded pupil size and gaze data at 1000 Hz. At FUB, the recording was monocular, and at UDE, it was binocular due to different licenses. We analyzed only the dominant eye for each participant. The centroid pupil-detection mode was used, with all heuristic filters switched off. The EyeLink was calibrated using a five-point calibration, with subsequent five-point validation requiring gaze accuracy below $1^{\circ }$ at each point. Pupil size was recorded as area in pixels, converted to diameter, and then to millimeters using setup-specific conversion factors derived from synchronized video frames (calibration procedure described below). Note that the millimeter conversion was done for visualization purposes and is not required for blink detection or duration estimation. The gaze signal was used only for excluding blinks when participants did not look near the target before and after the blink, see Section “Blink selection/quality criteria”.

#### Eyelid/eye openness tracking

**Fig. 1:**
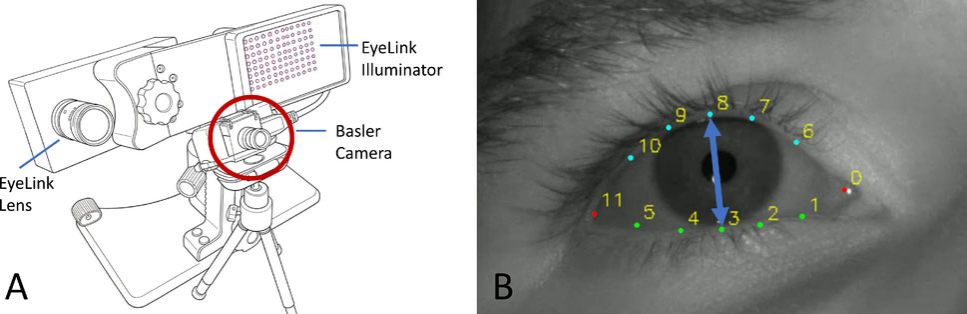
Experimental setup and eye openness estimation approach. (**A**) Recording setup: Synchronized data collection using the EyeLink 1000 Plus eye tracker for pupil-based measurements and a Basler high-speed camera (mounted on a mini tripod) for eyelid tracking. Both systems recorded simultaneously to enable comparison of blink detection methods. (**B**) Landmark detection: Example eye frame with 12 eyelid landmarks – five per eyelid and one at each canthus (inner and outer eye corners). Eye openness is the Euclidean distance between the central upper and lower eyelid landmarks (points 3 and 8; connected by the blue line), converted to millimeters via offline calibration

Eye openness was estimated by detecting eyelid landmarks in high-speed video recordings using a deep-learning model (Fig. 1). Recordings were obtained with a Basler monochrome high-speed camera (daA1920-160um; 16-mm lens; IR pass filter) at 500 Hz with $1088\times 320$ px resolution. Video was captured at 8-bit resolution with custom software (Hooge et al., 2021, 2024; Nyström et al., 2023), which streamed frames to mp4 format using libavcodec (ffmpeg v5.0) and the libx264 encoder (preset: veryfast, crf: 10, pixel format: gray). Illumination was provided by the EyeLink IR illuminator.

Eye regions were cropped from each frame (16:10 ratio), resized to $160\times 160$ px, and analyzed with a modified PIPNet model (Jin et al., 2021). We adapted the model architecture to detect 12 eyelid landmarks – five per eyelid and one at each canthus (inner and outer eye corners). The model was pretrained by us on a separate, custom dataset of 4200 curated eye images from 21 participants (distinct from the current study), balanced across openness states. Full details on training procedures and training dataset construction are provided in the Appendix A1.

Eye openness was defined as the Euclidean distance between the central upper and lower eyelid landmarks and converted to millimeters using an offline calibration procedure that involved recording a measurement tape placed at eye position to establish the pixel-to-millimeter conversion ratio (approximately 8 pixels per millimeter). Figure 1 illustrates the experimental setup and landmark-based eye openness measurement approach.

**Fig. 2:**
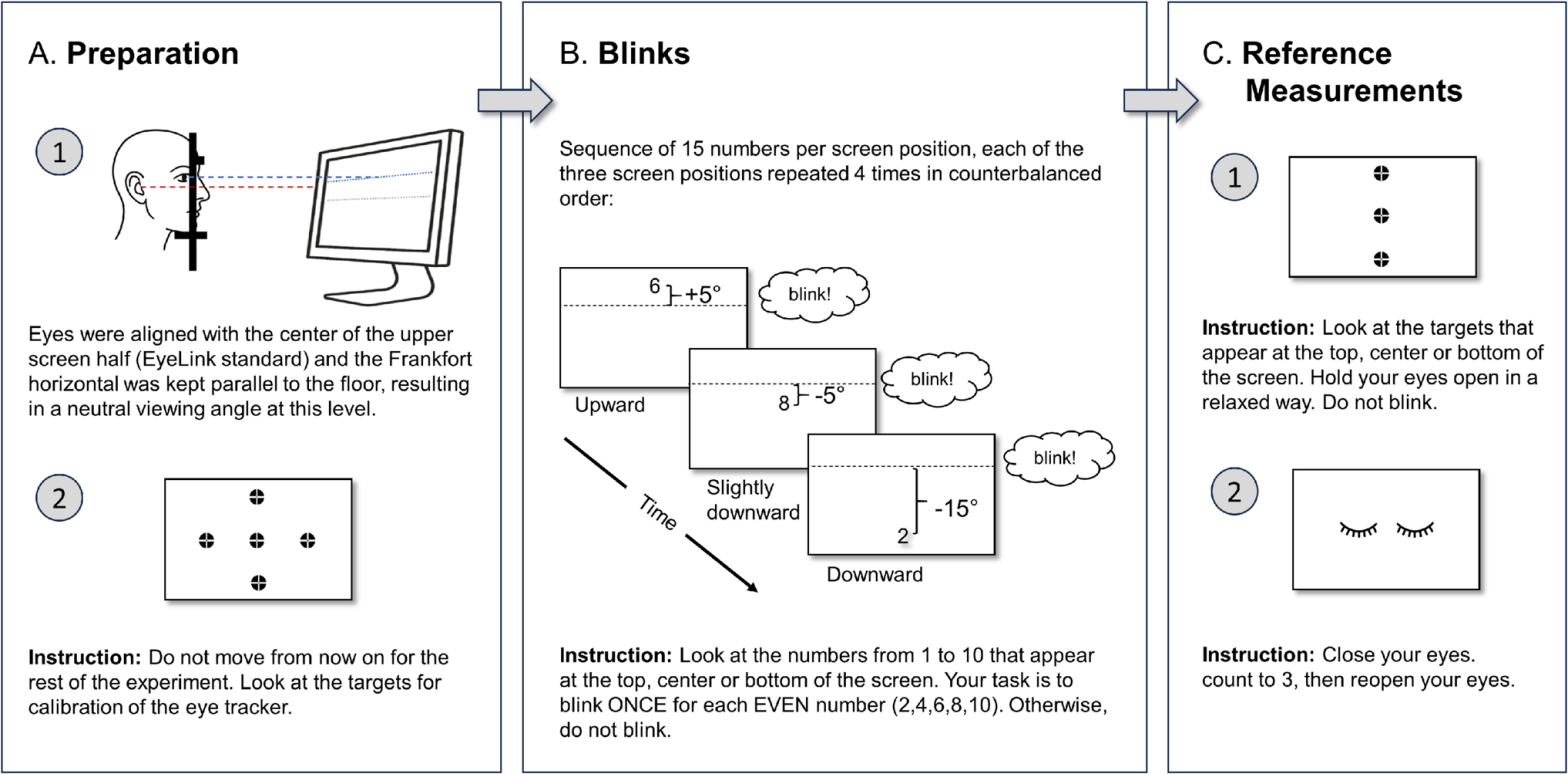
Experimental protocol. Setup and tasks for estimating blink parameters across vertical eye-in-head orientations. (**A**) Preparation. First, participants were positioned with the Frankfort horizontal plane (*red dashed line*) aligned parallel to the floor, establishing anatomically standardized head orientation. Eye height was aligned with the center of the upper screen half (i.e., neutral eye orientation at standard EyeLink configuration; *blue dashed line*). Second, the eye tracker was calibrated using a five-point calibration grid. (**B**) Voluntary blink task: Three vertical eye orientations, separated by 10$^{\circ }$, were tested. Participants completed 12 blocks (four per orientation), each with 15 trials at a single target position. Participants were instructed to blink after even numbers. Block order was randomized per participant without consecutive repeats of the same orientation. (**C**) Reference measurements: Two reference conditions assessed signal quality: First, maximum eye openness at each target position to calculate precision during steady fixation, and second, sustained eye closure to calculate precision during the closed state and confirm complete eyelid closure capability across participants

### Procedure

#### Setup and design

Participants were positioned with a chin-and-forehead rest at a 75-cm eye-to-screen distance. Head positioning followed a standardized protocol using the Frankfort horizontal plane, an established anatomical reference line from the eye socket to ear canal that defines neutral head posture in medical studies (Garson, 1885; Kollmann et al., 1884). Aligning this plane parallel to the ground ensured that vertical eye orientations were comparable across participants, preventing individual differences in head tilt from confounding eye-in-head measurements. Eye height was aligned with the center of the upper screen half, consistent with standard EyeLink procedures (SR Research, 2017, p. 66). Please note that gaze is the sum of eye and head orientation, but we are referring here to eye orientation with respect to the head. We achieve this by keeping the head in a fixed position and orientation, so that changes in gaze toward a different target position reflect only changes in eye orientation. Participants were explicitly instructed to maintain continuous contact with both the chin and forehead supports throughout the recording (i.e., not to lift from either support).

The experiment employed a within-subjects design with three target positions at vertical eye orientations of +5$^{\circ }$ (upward), −5$^{\circ }$ (slightly downward), and −15$^{\circ }$ (downward) relative to the head.

#### Experimental protocol

The experimental session consisted of three phases (Fig. 2). In the *preparation phase*, participants’ heads were positioned as previously described. Following successful calibration, participants completed a brief practice session to familiarize themselves with the task. During the *voluntary blink task*, participants completed 12 blocks (four blocks per position). Each block contained 15 trials at a single target position, for 180 trials in total (3 positions $\times$ 4 blocks per position $\times$ 15 trials per block). The order of the 12 blocks was randomized for each participant, with the constraint that the same target position did not appear in consecutive blocks. On each trial, a number (1–10) appeared at one of the three target positions for a jittered duration of 2–3 s. Eighty percent of the numbers were even and 20% were odd, yielding 48 even and 12 odd trials at each target position (60 trials per position). Participants were instructed to blink once when viewing even numbers and to refrain from blinking for odd numbers. This task was chosen to enhance participant engagement. There were no consecutive odd numbers to avoid prolonged blink suppression while maintaining task attention. Cued blinks were used to elicit a sufficient and comparable number of blinks at each controlled vertical eye orientation (i.e., target position) while keeping head orientation fixed. Finally, in the *reference measurement phase*, two reference conditions were collected to assess eye openness signal precision across different eye states: (1) maximum eye openness – participants held their eyes open in a relaxed manner while fixating each of the three target positions sequentially, providing precision values during steady fixation, and (2) complete closure – participants held their eyes closed in a relaxed manner for 3 s, providing precision values for the closed state and confirming that complete eyelid closure was achieved across participants.

### Data processing

#### Signal quality assessment

Precision was assessed using root mean square of sample-to-sample distances (RMS-S2S with a 100-ms sliding window; Hooge et al., 2018). Eye openness RMS-S2S ranged from 0.043 mm (open state) to 0.053 mm (closed state). Pupil diameter RMS-S2S was 0.008 mm during open eye states and 0.0004 mm when eyes were closed.

To assess eye openness accuracy, we conducted manual annotation on a subset of data. We randomly selected three blinks per participant (one from each target position) and extracted five frames during the blink transition. We additionally selected three additional frames per participant from the eyes-closed phase of the reference measurements. This yielded 18 frames per participant (306 frames total across all participants). Two independent raters manually annotated upper and lower eyelid positions for all frames. We assessed reliability using intraclass correlation coefficients (ICC (3,1)). Inter-rater agreement was excellent (ICC = 0.992; mean absolute error = 0.43 mm). Agreement between human raters and the landmark detection model was similarly strong (ICC = 0.986–0.988; mean absolute error = 0.80–0.81 mm), confirming excellent reliability (Koo & Li, 2016).

#### Blink detection

Blinks were detected using the algorithm described by Nyström et al., (2024), applied independently to both pupil diameter and eye-openness signals. The algorithm employed a two-stage process: (1) initial blink identification using prominent peak detection with minimum blink width of 15 ms at 50% relative height, and (2) refinement of onset and offset boundaries by tracing backward and forward from peak velocities until velocity dropped below thresholds of 3 $\times$ median absolute deviation (MAD) for eye openness and 10 mm/s for pupil diameter.

#### Blink selection/quality criteria

After each target number, one voluntary blink was selected for analysis from each signal (eye openness and pupil diameter). To ensure the blink corresponded to the intended eye orientation, the following criteria were applied:

- The distance between gaze point and target positions before and after each blink was $<{2}^{\circ }$ vertically and $<{3}^{\circ }$ horizontally.
- If multiple blinks occurred, only the first was retained, provided no subsequent blink occurred within 100 ms.
- Blinks were excluded if, between blink onset and offset, the pupil signal transiently recovered to $\ge {80}\%$ of its pre-blink diameter (indicative of a double blink).
- Selected blinks had to be present in both eye openness and pupil diameter signals, and the two had to overlap in time.

### Dependent variables

We extracted six primary dependent measures.

#### From pupil diameter signal (pupil-based method)

 

- *Pupil-based blink duration*: Blink duration determined from pupil disappearance and recovery.

#### From eye openness signal (eyelid-based method)

 

- *Eyelid-based blink duration (ms)*: Blink duration derived from eyelid movement (onset–offset).
- *Openness at onset (mm)*: Baseline eye openness before blink initiation.
- *Closing amplitude (mm)*: Maximum change in eye openness during closure.
- *Peak closing velocity (mm/s)*: Maximum downward velocity during closing phase.
- *Peak opening velocity (mm/s)*: Maximum upward velocity during the opening phase.

### Statistical analysis

Statistical analyses were conducted in R (version 4.3.2, R Core Team, 2023). For testing the influence of vertical eye orientation on estimated blink parameters, linear mixed-effects models were fitted separately for each dependent variable using the lme4 package (version 1.1.35.1, Bates et al., 2015), with a fixed effect for target position and a random intercept for each participant to account for individual differences in baseline values of the blink parameters. Estimated marginal means and contrasts were computed using the emmeans package (version 1.11.0, Lenth, 2025).

**Table 1:** Mean blink count after quality-based selection. *Blink-cue targets* are the even-number targets at each eye orientation (requesting a blink). *Selected blinks* are those retained after applying eye orientation accuracy criteria, temporal separation, and artifact exclusion. The *retention rate* is the proportion of blink-cue trials yielding valid blinks and thus also reflects how well participants followed instructions. Values are means (M) and standard deviations (SD) across 17 participants; $N=2{,}306$ valid blinks were included in subsequent analyses

Eye orientation	Blink-cue targets	Selected blinks (M ± SD)	Retention rate % (M ± SD)
+5$^{\circ }$ (upward)	48	46.69 ± 1.49	97.27 ± 3.11
–5$^{\circ }$ (slightly downward)	48	46.81 ± 2.43	97.53 ± 5.06
–15$^{\circ }$ (downward)	48	44.75 ± 4.73	93.23 ± 9.85

**Fig. 3:**
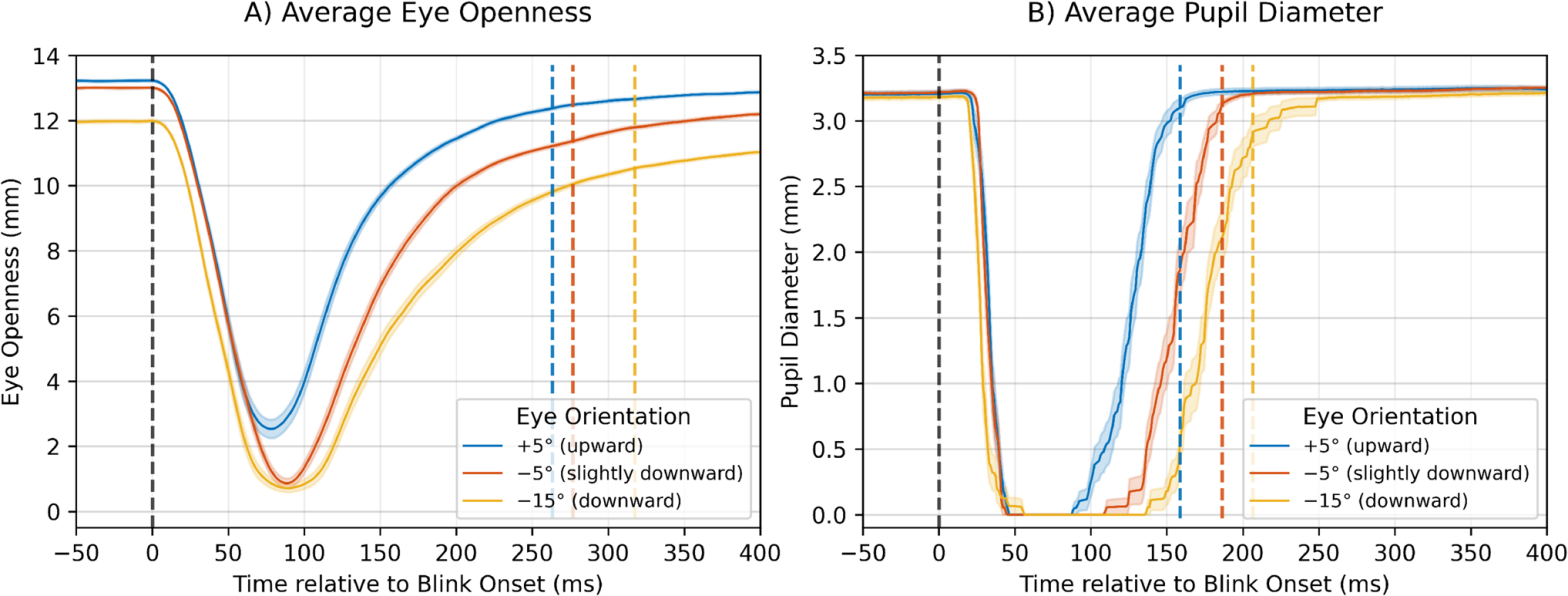
Blink waveforms. Average eye openness and pupil diameter waveforms by vertical eye-in-head orientation for a representative participant. (**A**) Eye openness grand averages (mean ± SEM) aligned to eye-openness-based blink onset ($t=0$). With more downward eye orientations, eyes are less open at blink onset, the overall blink duration is longer (rightward shift of the colored dashed offset line), and the reopening is slower (i.e., a flatter slope after peak closure). (**B**) Pupil-diameter grand averages (mean ± SEM), also aligned with the onset of blinking based on eye openness. For more downward eye orientations, there is a longer duration of complete pupil occlusion and a longer overall duration. *Colored dashed vertical lines* mark the average blink offset time for each eye orientation

## Results

### Blink counts

Table 1 summarizes blink counts by eye orientation. Across all 17 participants, the dataset contained $N=2306$ valid blinks (mean per participant $=135.65$; overall retention $\approx 96\%$). The number of selected blinks was similar across eye orientations, with slightly fewer retained for the downward (M = 44.75) compared to the slightly downward (M = 46.81) and upward (M = 46.69) eye orientations.

**Fig. 4:**
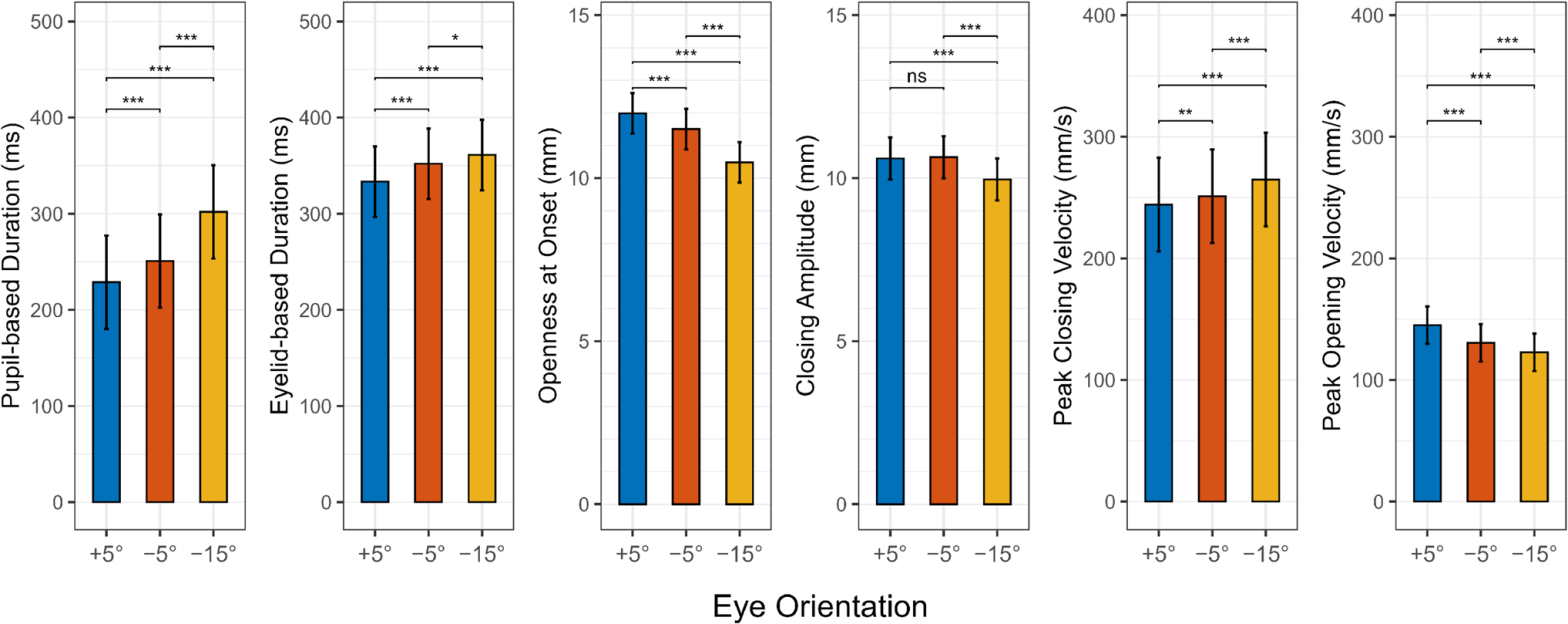
Blink parameters. Estimated marginal means by vertical eye-in-head orientation from linear mixed-effects models (with a fixed effect of eye orientation and random intercepts for participants). *Error bars* show 95% confidence intervals. Colors encode eye orientation (Upward (+5$^{\circ }$), Slightly downward (-5$^{\circ }$), Downward (-15$^{\circ }$)). *Horizontal brackets* indicate within-panel pairwise comparisons (Holm-adjusted): * $p<.05$, ** $p<.01$, *** $p<.001$. *Y*-axes share the same scale within units (mm, ms, mm/s) and start at zero. The linear mixed-effects models are based on $N=2,306$ blinks from 17 participants

### Effects of vertical eye orientation on estimated blink parameters

Average eye openness and pupil diameter waveforms from a representative participant illustrate the influence of vertical eye orientation (Fig. 3) on the estimated blink parameters. For eye openness, more downward eye orientations show both a longer total duration and a slower reopening (i.e., a flatter slope after peak closure). The pupil diameter waveform also shows a longer total duration, but seems to mainly reflect the longer pupil occlusion duration.

Mixed-effects models confirmed this pattern across participants (Fig. 4). All values reported below are estimated marginal means and contrasts from these models. Fixed-effect estimates are reported in the Appendix (Table 2). Vertical eye orientation systematically changed nearly all estimated blink parameters, with most effects aligning with our predictions based on findings of prior work using magnetic search coils. Exceptions were the closing amplitude and closing velocity, where we had predicted that upward eye orientation would consistently result in larger amplitudes and higher closing velocities. However, our results show no difference between upward (+5$^{\circ }$) and slightly downward (-5$^{\circ }$) eye orientation for closing amplitude. For closing velocity, our results are actually contrary to the prediction, as a lower closing velocity is shown with upward eye orientation.

#### Pupil-based blink duration

As expected, pupil-based blink durations were shortest for the upward eye orientation and longest for the downward eye orientation. Relative to the upward eye orientation (229 ms), blink durations increased by 22 ms for the slightly downward eye orientation and by 73 ms for the downward eye orientation (both $p < 0.001$), a $\sim 32\%$ prolongation from upward to downward that strongly supports our directional hypothesis.

#### Eyelid-based blink duration

Eyelid-based durations were consistent with our prediction of shorter durations with upward eye orientation, though with smaller effect magnitudes than observed for pupil-based detection. Compared to upward eye orientation (333 ms), blink durations were 19 ms longer at slightly downward and 28 ms longer at downward eye orientation (both $p < 0.001$), corresponding to a $\sim 8\%$ prolongation from upward to downward eye orientation.

#### Eye openness at onset

Eye openness at blink onset decreased monotonically with more downward eye orientation. This is consistent with previous findings that the upper eyelid starts at an elevated position during upward eye orientation. Compared to upward eye orientation (12.0 mm), openness was reduced by 0.5 mm at slightly downward ($p < 0.001$) and by 1.5 mm at downward eye orientation ($p < 0.001$).

#### Closing amplitude

We predicted larger closing amplitudes with upward eye orientation based on the elevated starting position of the upper eyelid, which was partially supported. Closing amplitude was similar for upward and slightly downward eye orientation ($p > 0.05$) with a mean of 10.6 mm, but decreased significantly for downward eye orientation by 0.6 mm relative to upward eye orientation ($p < 0.001$).

#### Velocity

Regarding closing velocity, our hypothesis predicted higher velocities with upward eye orientation based on prior magnetic search coil findings, but the opposite pattern emerged. Peak closing velocity increased in magnitude from 244 mm/s (upward eye orientation) to 265 mm/s (downward eye orientation), representing faster closing by 21 mm/s ($p < 0.001$). This finding contradicts our prediction based on the results of Vanderwerf et al. (2003). While peak closing velocity was higher for downward eye orientation, peak opening velocity decreased from 145 mm/s to 123 mm/s over the same range, a reduction of 22 mm/s ($p < 0.001$). While we had no directional prediction based on previous research, the observed pattern of slower opening with downward eye orientation is consistent with the overall trend toward prolonged blink dynamics at downward eye orientations.

### Method-dependence of the sensitivity of blink duration to vertical eye orientation

Both pupil-based and eyelid-based measures revealed that vertical eye orientation substantially altered blink duration, yet the magnitude of change differed substantially between the two signals. Pupil-based blink duration changed by approximately 32% ($+73$ ms) across the tested vertical eye orientations, whereas eyelid-based blink duration changed by only approximately 8% ($+28$ ms).

This discrepancy in sensitivity to vertical eye orientation raises an important consideration: different studies employ different methods to determine blink onset and offset from eyelid or pupil signals, and these methodological choices could themselves contribute to varying sensitivities to vertical eye orientation. To assess whether the method used to determine blink duration affects sensitivity to vertical eye orientation, we asked: *Does the choice of method to determine blink onset and offset contribute to differences in sensitivity?* This analysis serves to contextualize the substantially different results from pupil-based and eyelid-based measures within the broader landscape of blink analysis methods used across the literature.

In the present study, we used a velocity-based algorithm for the eyelid-based blink duration (in the spirit of “Lid Quiescence” Stern et al., 1984), as implemented by Nyström et al., (2024). To explore whether the sensitivity to vertical eye orientation changes when using a different method to derive blink duration, we recalculated eyelid-based blink duration using a 50% amplitude criterion (defining onset and offset at half of the closing and opening amplitudes), which is widely used in the blink analysis literature (e.g., Anund et al., 2017; Kleifges et al., 2017; Filtness et al., 2014; Picot et al., 2009; Bardouille et al., 2006).

This alternative approach yielded blink duration estimates of 154 ms at upward eye orientation and 179 ms at downward eye orientation, corresponding to a change of approximately 16% ($+25$ ms) – closer to the pupil-based blink duration in both baseline value and sensitivity to vertical eye orientation. These findings indicate that the method used to determine blink onset and offset modulates the extent to which vertical eye orientation alters blink duration estimates. Importantly, all methods showed the same directional pattern: blink duration consistently increased with downward eye orientation. Consequently, the method used to derive blink duration not only determines baseline duration values but also the sensitivity to vertical eye orientation.

## Discussion

### Summary of results

We investigated whether vertical eye-in-head orientation influences blink parameters estimated with modern video-based methods, and whether this influence differs between eyelid-based and pupil-based detection. Across 2306 voluntary blinks from 17 participants, vertical eye orientation systematically affected nearly all blink parameters.

Relative to an upward eye orientation ($+{5}^{\circ }$), a downward eye orientation ($-{15}^{\circ }$) was associated with (i) smaller eye openness at onset ($-1.5$ mm), (ii) a slight reduction in closing amplitude ($-0.6$ mm), (iii) longer eyelid-based blink duration ($+28$ ms; $\sim \!8\%$), and (iv) slower peak opening velocity ($-22$ mm/s). Moreover, peak closing velocity was higher at more downward orientations (+21 mm/s).

This may appear counterintuitive, because starting from a more closed position (and showing a slightly reduced closing amplitude) might be expected to shorten blinks. However, blink duration reflects the combined timing of the closing phase, any time spent near full eye closure, and the reopening phase. In our data, longer blink durations at more downward eye orientations are consistent with the pronounced slowing of reopening (reduced peak opening velocity). Moreover, peak closing velocity increased while peak opening velocity decreased with more downward orientation, further suggesting that the prolongation reflects changes in reopening rather than slower closing.

Pupil-based blink duration showed the same qualitative pattern but with a larger magnitude: relative to upward eye orientation, blink durations increased by 73 ms at downward eye orientation, i.e., a $\sim \!32\%$ prolongation. Since pupil-based duration depends on the visibility of the pupil (i.e., when the pupil can be detected by the eye tracker), slower reopening can cause a delay until the pupil becomes visible again, thereby also extending the estimated pupil occlusion interval.

The sensitivity of blink duration to vertical eye orientation also depended on the method used to determine blink onset and offset: recalculating eyelid-based blink duration using a 50% amplitude criterion yielded $\sim \!16\%$ longer blink durations, intermediate between the $\sim \!8\%$ increase (eyelid-velocity) and the $\sim \!32\%$ increase (pupil-based).

Taken together, vertical eye orientation is a relevant source of systematic variation in video-based blink parameters, with effects on blink duration being especially pronounced for pupil-based measurements. Because blink duration is arguably the most widely used blink metric in cognitive, clinical, and applied work (e.g., Caffier et al., 2003; Picot et al., 2009; Benedetto et al., 2011; Hollander and Huette, 2022; Oyarzo et al., 2022), this is practically important for interpretation and comparability across studies.

Because we used cued voluntary blinks, it remains to be tested whether the vertical eye orientation effects generalize to spontaneous blinks during more natural viewing with continuous monitoring of vertical eye orientation. Future work could further include systematic changes in horizontal eye orientation, despite previous studies showing no effect in this dimension (Vanderwerf et al., 2003).

### Comparisons with search-coil studies

Several of our findings align with magnetic search-coil studies, as predicted: upward eye orientation is associated with a more open eye at blink onset and with shorter blinks, whereas downward orientation yields longer blinks and reduced amplitude (Stern et al., 1984; Vanderwerf et al., 2003). Vanderwerf et al. (2003) reported shorter total blink duration at upward eye orientation (i.e., 121 ms to 163 ms) compared to downward (i.e., 154 ms to 203 ms), along with larger closing amplitudes and higher maximal closing velocities at upward orientation. Using non-invasive video-based measurements and smaller eye orientation changes, our eyelid-based blink durations replicate the directional blink duration pattern reported by Vanderwerf et al. (2003) (shorter at upward, longer at downward orientations).

Unlike Vanderwerf et al. (2003), who reported higher peak closing velocities at more upward eye orientations, we observed higher peak closing velocities at more downward eye orientations. With the present data, we cannot determine the reason for this discrepancy. We note, however, several differences between studies that could contribute to different findings: (i) the range of tested eye orientations (approximately $\pm {30}^{\circ }$ in (Vanderwerf et al., 2003) vs. $+{5}^{\circ }$, $-{5}^{\circ }$, $-{15}^{\circ }$ here), (ii) blink instructions (“blink as fast as possible” vs. “blink naturally”), (iii) measurement method (magnetic search coils vs. video-based tracking), and (iv) sample size (four vs. 17 participants).

Taken together, blink duration and amplitude results follow our predictions derived from Vanderwerf et al. (2003), with similar direction and comparable magnitude, now shown in a larger sample using video-based measurements. The differences in blink duration for different vertical eye orientation was substantially larger for pupil-based than eyelid-based estimates. In contrast to our prediction, peak closing velocity increased with more downward eye orientation. Finally, our exploratory analysis showed that opening velocity decreased with more downward orientation – a measure not reported by Vanderwerf et al. (2003).

### Implications for research using blink measures

Vertical eye orientation is a confound for blink parameters. In our study, a 20$^{\circ }$ downward change of eye orientation increased blink duration by up to 73 ms ($\sim$32%) for pupil-based measures and 28 ms ($\sim$8%) for eyelid-velocity-based measures. These effect sizes match or exceed differences commonly attributed to cognitive or state factors in the literature – drowsiness (alert vs. drowsy $\approx$56 ms, $\sim$28%) (Caffier et al., 2003), time-on-task while driving ($\approx$15 ms, $\sim$10%) (Benedetto et al., 2011), and engagement/content manipulations in reading and audio tasks ($\approx$9 ms to 15 ms, $\sim$2% to 4%) (Hollander & Huette, 2022). Consequently, when vertical eye orientation is unconstrained or unmeasured, differences of this magnitude cannot be reliably attributed to cognitive factors. Small shifts in vertical eye orientation can inflate, attenuate, or mimic reported effects.

This poses an interpretability problem for common designs that likely induce systematic changes in vertical eye orientation between conditions – for example, comparing content at different vertical positions on a display or alternating between views that naturally differ in gaze elevation. If vertical eye orientation differs between conditions, this results in observed differences in blink duration, irrespective of any measured cognitive variable or intended cognitive manipulation.

This also affects the comparability and replicability of studies. Seemingly minor differences in the experimental setup – such as the screen height, the eye-to-screen distance, and head positioning – can shift the vertical eye orientation by a few degrees, which in turn can change reported blink durations, even in the absence of cognitive differences. In addition, the method used to derive blink duration (e.g., pupil-occlusion interval, eyelid kinematics with a velocity threshold, or a 50% amplitude criterion) influences both baseline blink duration and sensitivity to changes in vertical eye orientation.

Consequently, differences in blink duration of a small-to-moderate size should not be attributed to cognitive factors unless vertical eye orientation has been measured, controlled for, or shown not to differ between conditions.

### Recommendations for research using blink measures

Our findings motivate practical recommendations for blink research and applications:

1. *Quantify and document vertical eye orientation* relative to the head and document it in relation to the variable structure of the study design (e.g., document the mean, standard deviation, and range per condition).
2. *Design the study so that changes in vertical eye orientation are minimized.* Where eye orientation variation by task is necessary (e.g., reading from top to bottom; looking at the road vs. instruments), design conditions to minimize eye orientation differences (i.e., matching) or statistically account for vertical eye orientation (e.g., by adding it as a covariate). This recommendation parallels standard practice in pupillometry, where gaze-position dependencies of measured pupil size are well documented and typically controlled for in experimental designs (Mathôt et al., 2018; Mathôt & Vilotijević, 2022).
3. *Be explicit about the method to determine blink duration.* Specify how blink onset and offset are determined (e.g., velocity-thresholds, pupil-occlusion interval, or 50% amplitude) and, where feasible, report multiple methods with respective estimates, as the method to determine blink duration modulates the sensitivity to vertical eye orientation changes.

### Conclusion

Vertical eye orientation systematically influences video-based blink parameters. Changes in blink duration are particularly pronounced for pupil-based measures. Part of the difference in magnitude of vertical eye orientation sensitivity between pupil-based and eyelid-based methods reflects the different approach used to determine blink onset and offset. Studies and applications that investigate blink characteristics should record vertical eye orientation or control for it by design to improve the validity and comparability of blink research and its applications.

## Data Availability

Raw recordings unavailable due to privacy regulations. The anonymized aggregated dataset used for the analyses is available in the OSF repository (https://osf.io/tud8a).

## A1 Details of deep-learning model training for eyelid-/eye-openness tracking

We trained a PIPNet-based landmark detector (Jin et al., 2021) adapted for single-eye inputs to localize 12 eyelid landmarks (five upper, five lower, and one at each canthus). The landmark detector operated on frames cropped around a single eye and resized to $160\times 160$ px (grayscale). We trained this detector on a custom infrared eye-image dataset comprising 4200 manually curated single-eye images from 21 participants (distinct from those in the current study), sampled to cover the full range of eye openness from fully closed to widely open.

Eyelid landmarks for the dataset were annotated with a custom labeling tool. To maximize accuracy for challenging cases (e.g., eyelashes occluding a nearly closed eye), human annotators could step through neighboring frames to track eyelid motion. For each eyelid, the annotator traced the lid with the mouse; a polynomial curve (2nd–4th degree) was then fitted between the medial and lateral canthi. From the line connecting these canthi, five equally spaced perpendiculars intersected the segmentation boundary to define five landmark points per eyelid. Eye openness was computed as the Euclidean distance between the central upper and central lower landmarks; when the eye was closed, a single line yielded coincident upper and lower landmarks.

We included left and right eyes, but right-eye images were horizontally flipped, so the landmark detector had to learn only on eye images with the inner canthus (i.e., eye corner) on the left side. For evaluation, we held out 100 images per subject as a subject-specific test set. The remainder was split 80 %/20 % into training/validation. To assess annotation quality, 50 % of the test set (50 images per subject) was independently annotated by a second annotator; inter-rater agreement was high across eye openness levels (mean absolute error, MAE = 0.3127 mm; Pearson’s $r={0.9916}$).

Data augmentation during training followed the original PIPNet procedures. Random in-plane rotations in the range $-15^{\circ }-{15}^{\circ }$ were applied to 30 % of images, with additional random translations along $x$ and $y$. When rotations/translations were applied, the new frame areas were extracted again from the original video frames, so that no padding was necessary. Images were normalized, and contrast-limited adaptive histogram equalization (CLAHE; clip limit 1.5, tile grid ${2}\times {2}$) was applied.

Neighborhood-regression used two neighboring landmarks, and landmark priors were set to the average configuration of an eye opened by 5 mm. Training ran for 60 epochs (batch size 32); the learning rate was initialized at 1e-4 and decayed at epochs 30 and 50. Losses followed Jin et al. (2021) (MSE for classification, L1 for regression). The backbone was initialized with ImageNet-pretrained weights. Validation loss was monitored; as it consistently converged under this fixed schedule (matching the original authors), model selection did not rely on early stopping. Hyperparameters followed the original work with a small informal search to confirm stability on our dataset.

## A2 Fixed effects from linear mixed-effects models

**Table 2:** Fixed effects from linear mixed-effects models. Separate models were fit for each blink parameter with vertical eye orientation as a fixed effect and random intercepts for participants: $\textit{DV} \sim \textit{orientation} + (1|\textit{participant})$. Orientation was reference-coded with upward (+5$^{\circ }$) as the reference level; the intercept therefore corresponds to the estimated mean for the upward condition. Degrees of freedom and *p*-values are from Satterthwaite’s approximation (lmerTest), and may vary across fixed-effect terms; 95% confidence intervals are Wald intervals. Models are based on $N=2306$ blinks from 17 participants

Parameter	Fixed effect	*b*	SE	*df*	*t*	*p*	95% CI
Openness at onset (mm)							
	+5$^{\circ }$ (Intercept)	12.0	0.287	17.1	41.8	$<.001$	[11.4, 12.5]
	-5$^{\circ }$ vs +5$^{\circ }$	-0.484	0.0289	2289	-16.7	$<.001$	[-0.540, -0.427]
	-15$^{\circ }$ vs +5$^{\circ }$	-1.50	0.0292	2289	-51.4	$<.001$	[-1.56, -1.45]
Closing amplitude (mm)							
	+5$^{\circ }$ (Intercept)	10.6	0.298	17.5	35.5	$<.001$	[10.0, 11.2]
	-5$^{\circ }$ vs +5$^{\circ }$	0.0343	0.0616	2289	0.557	.577	[-0.0864, 0.155]
	-15$^{\circ }$ vs +5$^{\circ }$	-0.646	0.0623	2289	-10.4	$<.001$	[-0.768, -0.524]
Peak closing velocity (mm/s)							
	+5$^{\circ }$ (Intercept)	244	17.8	17.2	13.7	$<.001$	[209, 279]
	-5$^{\circ }$ vs +5$^{\circ }$	6.87	2.23	2289	3.08	.002	[2.49, 11.2]
	-15$^{\circ }$ vs +5$^{\circ }$	20.6	2.26	2289	9.10	$<.001$	[16.1, 25.0]
Pupil-based duration (ms)							
	Intercept (upward +5$^{\circ }$)	229	22.4	17.4	10.2	$<.001$	[185, 273]
	-5$^{\circ }$ vs +5$^{\circ }$	22.1	4.09	2289	5.40	$<.001$	[14.1, 30.1]
	-15$^{\circ }$ vs +5$^{\circ }$	73.2	4.14	2289	17.7	$<.001$	[65.1, 81.3]
Eyelid-based duration (ms)							
	Intercept (upward +5$^{\circ }$)	333	17.0	17.5	19.6	$<.001$	[300, 367]
	-5$^{\circ }$ vs +5$^{\circ }$	18.6	3.62	2289	5.13	$<.001$	[11.5, 25.7]
	-15$^{\circ }$ vs +5$^{\circ }$	27.7	3.67	2289	7.57	$<.001$	[20.6, 34.9]
Peak opening velocity (mm/s)							
	Intercept (upward +5$^{\circ }$)	145	7.13	17.4	20.3	$<.001$	[131, 159]
	-5$^{\circ }$ vs +5$^{\circ }$	-14.5	1.31	2289	-11.0	$<.001$	[-17.1, -11.9]
	-15$^{\circ }$ vs +5$^{\circ }$	-22.3	1.33	2289	-16.8	$<.001$	[-24.9, -19.7]

## Code availability

The Python and R code used for data processing and analyses are available at https://osf.io/tud8a.
